# Supplementary material for: Dynamic enlargement and mobilization of lipid droplets in pluripotent cells coordinate morphogenesis during mouse peri-implantation development
Source: Nat Commun. 2022 Jul 5;13:3861. doi: 10.1038/s41467-022-31323-2 (PMC9256688; doi:10.1038/s41467-022-31323-2)
Supplement: Supplementary file 1 — Supplementary Information [file 41467_2022_31323_MOESM1_ESM.pdf]

Dynamic Enlargement and Mobilization of Lipid Droplets in Pluripotent Cells Coordinate Morphogenesis during Mouse Peri-implantation Development

Mau *et al.*

Supplementary Information

Supplementary Figure 1, *Cidea* is highly induced in embryonic stem cells cultured under lipid-rich conditions.

Supplementary Figure 2, Enforcing or abolishing *Cidea* expression alters the behaviour of LDs without affecting the undifferentiated status of ESCs.

Supplementary Figure 3, CIDEA is strictly required to fulfil the full process of LD enlargement in ESCs.

Supplementary Figure 4, Enlarged LDs accumulated in the blastocyst are mobilized upon implantation in the epiblast lineage.

Supplementary Figure 5, CIDEA knockout ESCs acquire normally a primed pluripotent state in 3D cultures.

Supplementary Figure 6, CIDEA knockout disrupts lumenogenesis in pluripotent cells *in vitro* and *in vivo*.

Supplementary Figure 7, Lysosomal degradation of LDs is specifically required for spheroid morphogenesis.

Supplementary Figure 8, Lipophagy coordinates the timely mobilization of LDs and morphogenesis during peri-implantation development.

Supplementary Figures

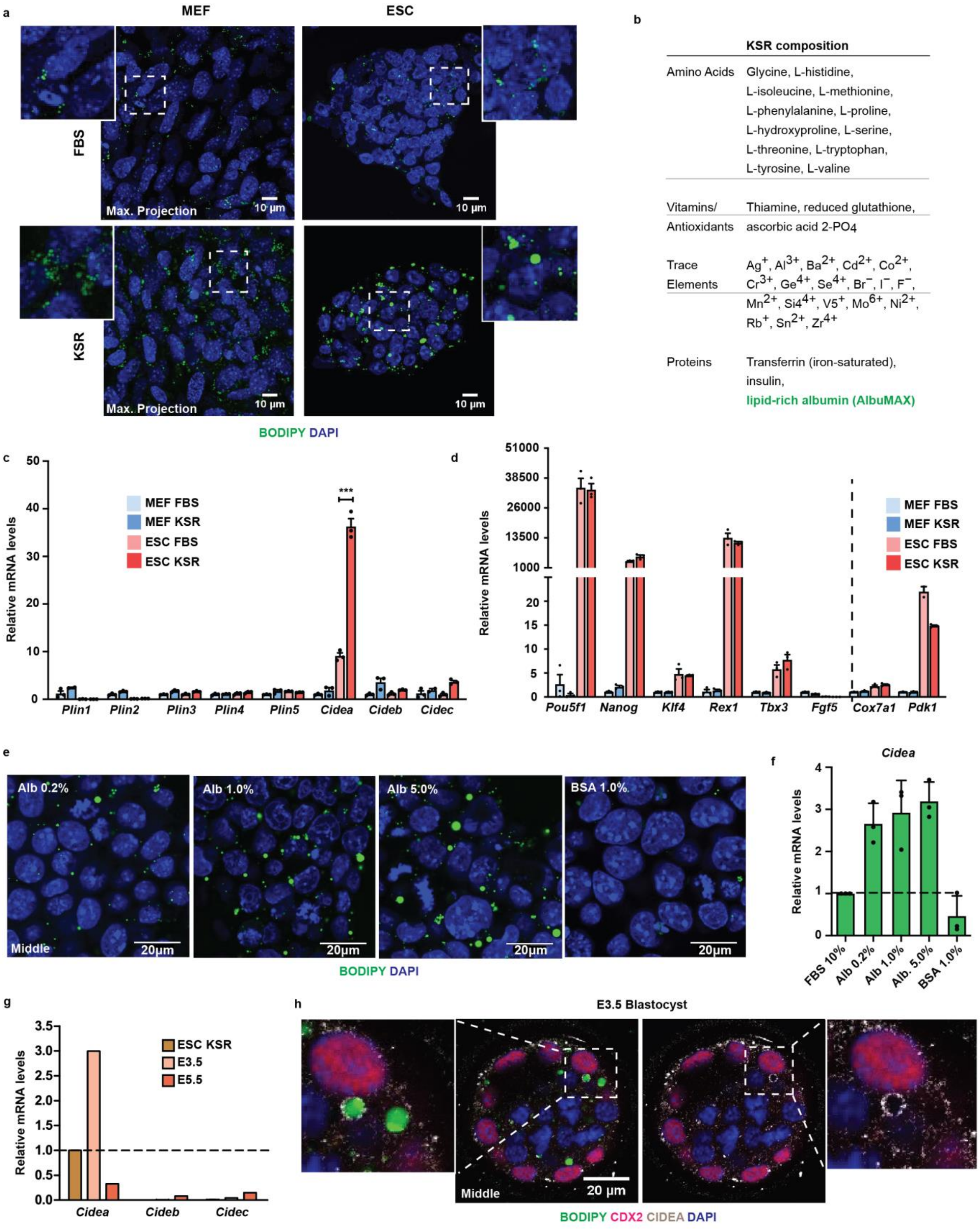

Supplementary Figure 1 | *Cidea* is highly induced in embryonic stem cells cultured under lipid-rich conditions. (a) Representative images of BODIPY 493/503-stained LDs in MEFs and E14-ESCs cultured in foetal bovine serum (FBS) and knockout serum replacement (KSR)-supplemented media (n=3). Squares indicate magnified regions. **(b)** Composition of KSR as previously reported[1]. **(c,d)** Expression (RT-qPCR) of perilipin (*Plin*) and cell death

inducing DFF45-like effector (*Cide*) gene family members (c), pluripotency-associated *Pou5f1* (also known as *Oct4*), *Nanog*, *Klf4*, *Rex1* (also known as *Zfp42)*, *Tbx3* and *Fgf5* (left), and metabolic *Pdk1* and *Cox7a1* (right) genes (d) in MEFs (blue) and E14-ESCs (red) cultured in FBS and KSR-supplemented media (see also Fig. 2c showing the same raw data for the *Cidea* transcript only). Data are expressed relative to MEFs (FBS = 1). Error bars, means ± s.e.m. (n=3); two-sided unpaired Student's t-test; ***, p<0.001 ($p=9.4x10^{-5}$). **(e)** Images of BODIPY-stained LDs in E14-ESCs cultured in media supplemented with increasing concentrations of AlbuMax (Alb) and lipid free BSA fraction V (BSA) used as control (n=3). **(f)** Expression (RT-qPCR) of *Cidea* transcript in cells from (e). Data are expressed relative to FBS samples (FBS =1; dotted line). Error bars, means ± s.e.m. (n=3). **(g)** Expression (RT-qPCR) of *Cidea*, *Cideb* and *Cidec* transcripts in E14-ESCs grown in KSR-supplemented medium and pools of freshly harvested E3.5 and E5.5 embryos (n=5-8). Data are expressed relative to ESC control (ESC KSR =1; dotted line); error bars, means ± s.e.m (n=3). **(h)** Representative images of BODIPY-labelled LDs and CIDEA protein detected at the surface of LDs by immunostaining using an anti-CIDEA antibody from Santa Cruz (sc-8730-R) in freshly harvested E3.5 blastocysts (n=6). CDX2, trophectoderm lineage marker. Source data are provided as a Source Data file.

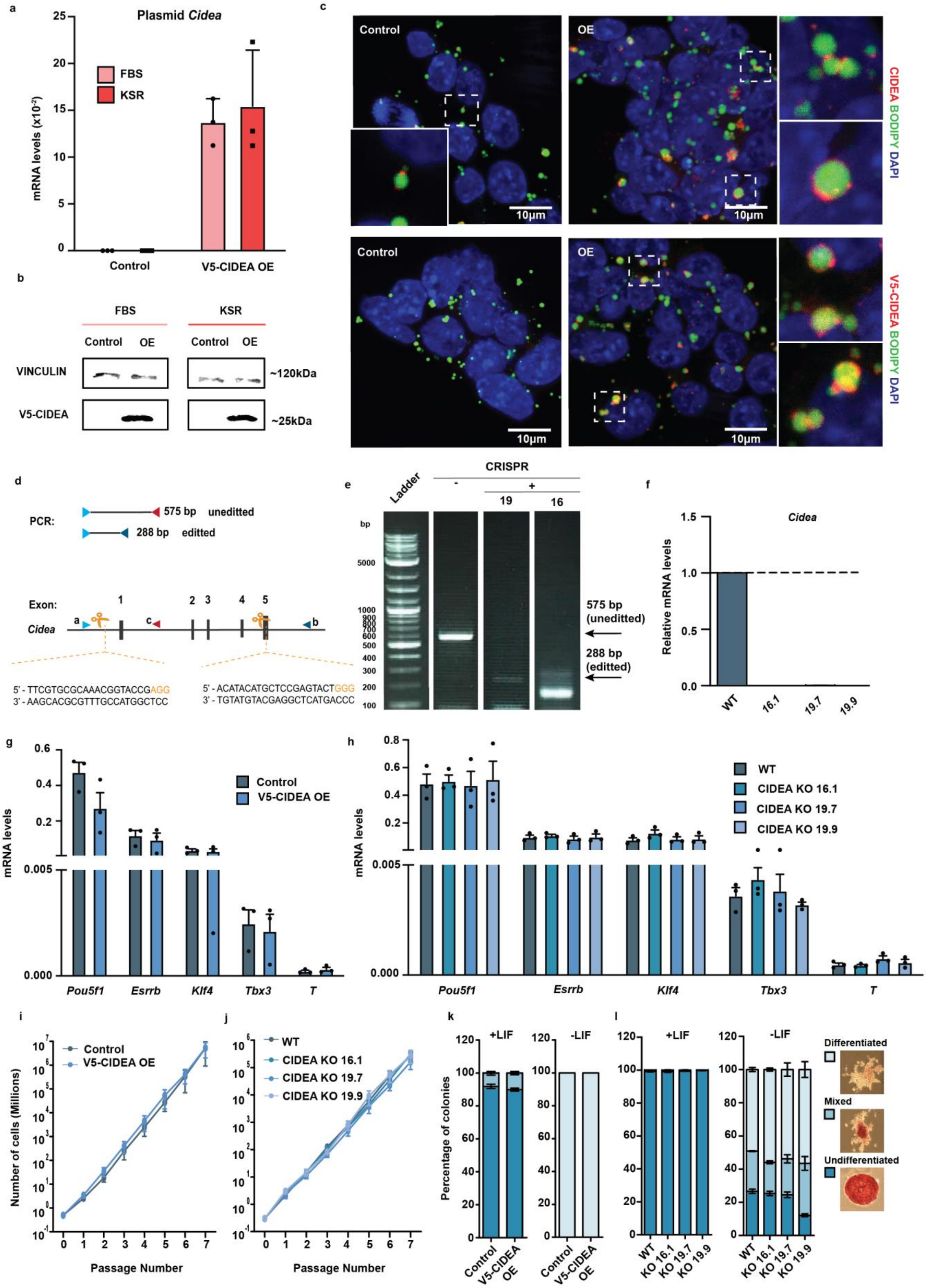

Supplementary Figure 2 | Enforcing or abolishing *Cidea* expression alters the behaviour of LDs without affecting the undifferentiated status of ESCs. (a) Expression of exogenous (plasmid) *Cidea* transcript (RT-qPCR) in stably transfected E14-ESCs with empty (control)

and V5-tagged *Cidea* (V5-CIDEA) constructs grown in FBS and KSR-supplemented media. Each symbol represents one biological replicate. Error bars, means ± s.e.m. (n=3). OE, overexpression. **(b)** Western blot analysis of V5 epitope and VINCULIN (loading control) in cells from (a) (n=2). **(c)** Immunostaining images of total (top) and exogenous (bottom) CIDEA protein at LD-LD contact sites and at the surface of enlarged LDs labelled by BODIPY 493/503 in control and V5-CIDEA OE E14-ESCs exposed to KSR (~ 6-7hours) (n=3). Squares indicate magnified regions. CIDEA protein was detected by immunostaining using an anti-CIDEA antibody from Proteintech (13170-1-AP). **(d)** Strategy of CRISPR-Cas9 deletion of the *Cidea* locus. Scissors indicate the position of sites targeted by gRNAs; arrows indicate the position of genotyping primers. Primers a and b amplify *Cidea* edited alleles, while primers a and c amplify unedited alleles, yielding 288bp and 575bp PCR products, respectively. **(e)** Genomic PCR analysis of homozygous CIDEA knockout (KO) ESC clones (16.1, 19.7 and 19.9) along with parental wild-type (WT) R1-ESCs used as control. **(f)** Expression (RT-qPCR) of *Cidea* transcript in CIDEA KO clones relative to WT ESCs grown in FBS-supplemented medium. Data are expressed relative to control samples (WT=1; dotted line). Error bars, means ± s.e.m. (n=3). **(g,h)** Expression (RT-qPCR) of pluripotency-associated genes (*Pou5f1*, *Esrrb*, *Klf4*, *Tbx3*) and early differentiation marker *T* (also known as *Brachyury*) in V5-CIDEA OE and CIDEA KO ESCs along with matching controls in FBS-supplemented medium. Each dot represents one biological replicate; error bars, means ± s.e.m. (n=3). **(i,j)** Growth curves of cells over 7 passages in FBS-supplemented medium; error bars, means ± s.e.m. (n=3). (**k,l)** Percentage of colonies formed from cells seeded at low density and grown for 7 days with (+) and without (-) LIF in FBS-supplemented medium. Colonies were counted and scored as undifferentiated, mixed, and differentiated based on the intensity of alkaline phosphatase staining. Error bars, means ± s.e.m. (n=3). Source data are provided as a Source Data file.

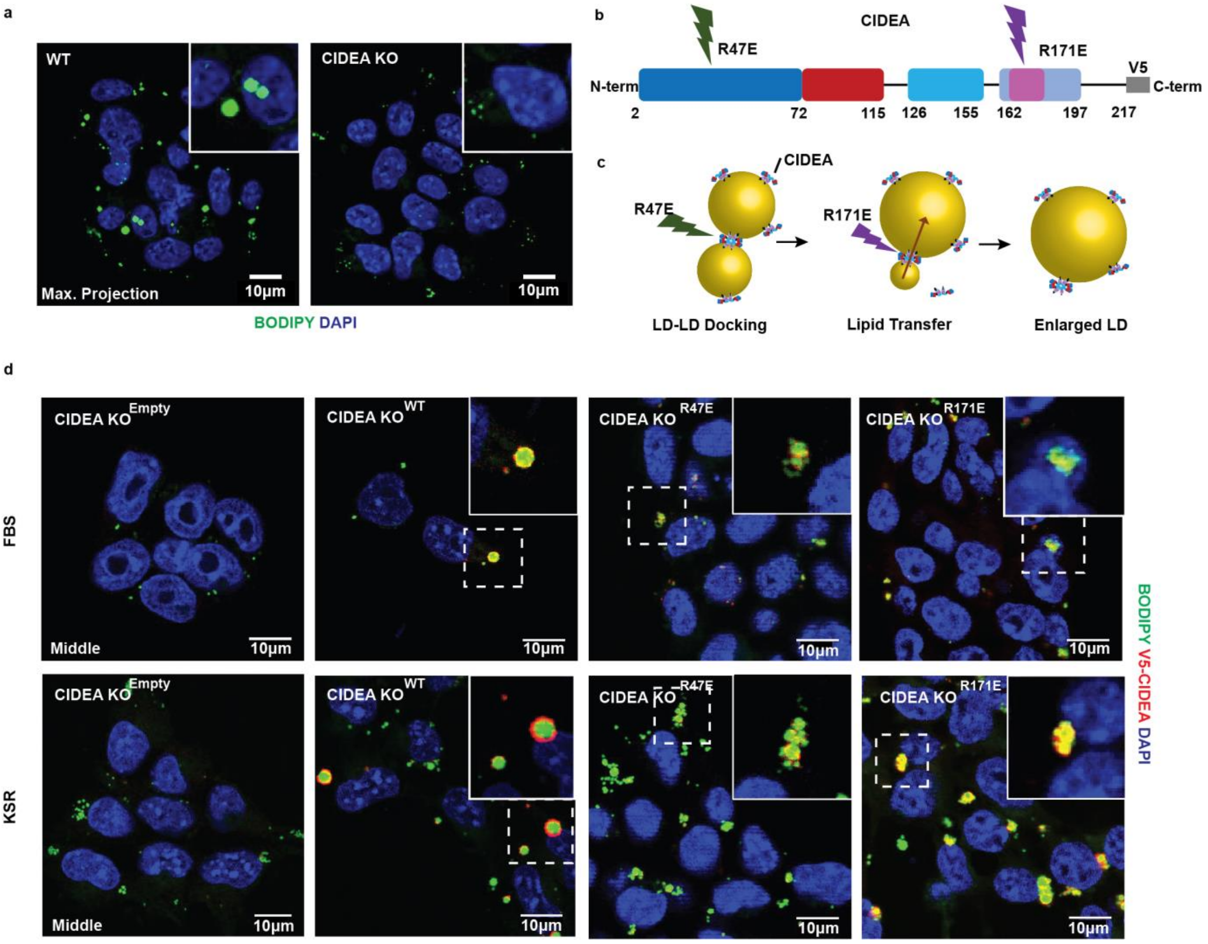

Supplementary Figure 3 | CIDEA is strictly required to fulfil the full process of LD enlargement in ESCs. (a) Representative images of BODIPY 493/503-stained LDs in parental WT and CIDEA KO R1-ESCs cultured in KSR-supplemented medium (clone 19.7). Similar data were obtained with clones 16.1 and 19.9 (n=3). **(b)** Scheme depicting V5-CIDEA protein with indications of point-mutation (R47E and R171E) insertion sites via substitution of Arginine (R) with Glutamate (E). The N-terminal of CIDEA consists of a basic region (2-72 amino acids) followed by an acidic region (73-110 amino acids). Its C-terminal is mostly basic and encompasses a highly conserved region amongst CIDE family members (126-155 amino acids) and a hydrophobic region (162-197 amino acids). Blue, basic; red, acidic; magenta, amphipathic; grey, V5, epitope. **(c)** Effects of CIDEA point-mutations (R47E and R171E) on the process of LD enlargement. CIDEA mediates its function on the surface of LDs where it promotes the docking of adjacent LDs by forming CIDEA trans-complexes through contributions of the N-terminal and a C-terminal dimerization region. These complexes, enriched at LD-LD contact sites, interact with LD phospholipid monolayer via an amphipathic helix and likely increase phospholipid barrier permeability, promoting LD fusion by transference of lipids as previously described[2]. Point-

mutation R47E within N-terminal region leads to LD-LD docking defect. Point-mutation R171E within amphipathic helix leads to lipid transfer defect. **(d)** Representative images of BODIPY stained-LDs and V5 epitope in CIDEA KO ESCs (clone 19.7) stably transfected with control (CIDEA KO^{Empty}), wild-type (CIDEA KO^{WT}) and point-mutated (CIDEA KO^{R47E} and CIDEA KO^{R171E}) V5-tagged *Cidea* constructs, in FBS- and KSR-supplemented media (n=3) (see also Fig. 2g showing the same images in KSR-supplemented medium only).

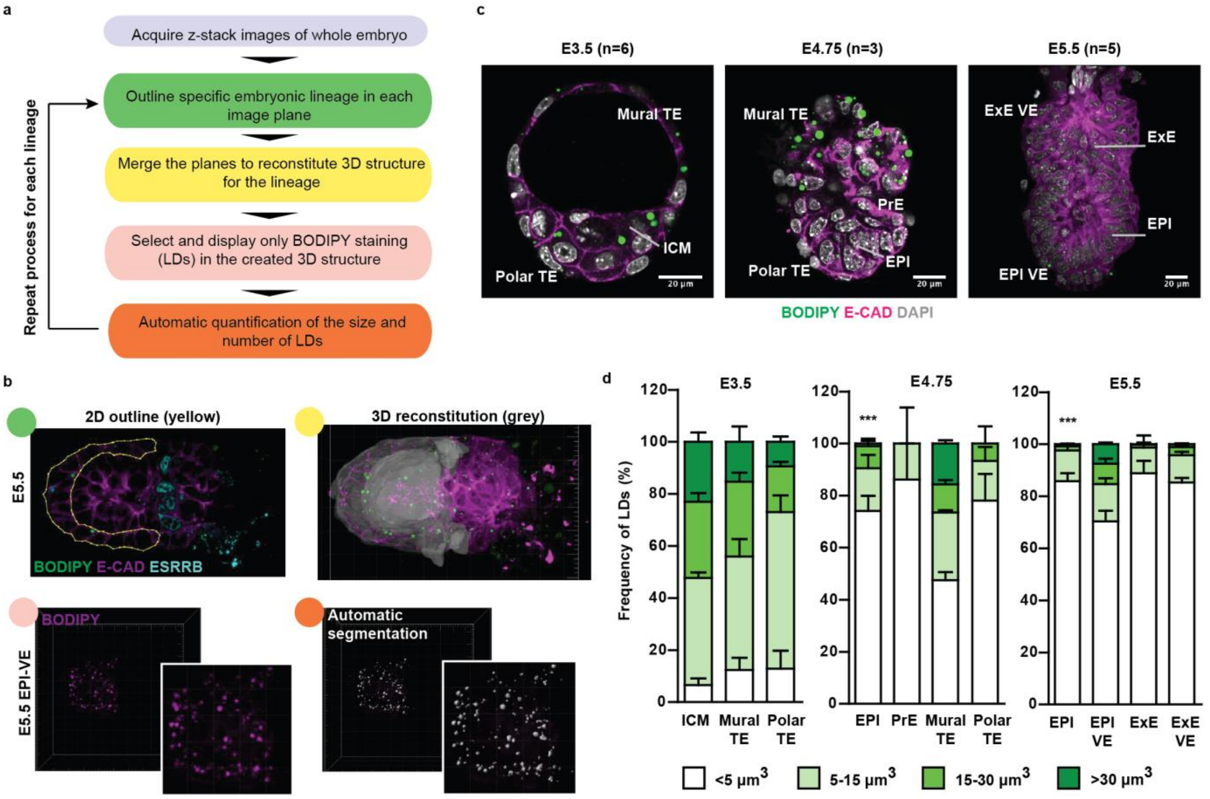

Supplementary Figure 4 | Enlarged LDs accumulated in the blastocyst are mobilized upon implantation in the epiblast lineage. (a) Schematic flow of image processing and automatic quantification of number and volume of LDs in a lineage-dependent manner. **(b)** Examples of image processing as outlined in (a). **(c)** Representative images of BODIPY 493/503-labelled LDs in freshly harvested E3.5 blastocysts, E4.75 and E5.5 peri-implantation embryos immunoassayed against E-CADHERIN (E-CAD) (n=3-6 embryos). ICM, inner cell mass; TE, trophectoderm; EPI, epiblast; PrE, primitive endoderm; ExE, extraembryonic ectoderm; VE, visceral endoderm. **(d)** Quantification of the volume (μm^3) and number of LDs in early blastocysts and peri-implantation embryos from (c). Error bars, means ± s.e.m. (n=3-6); Average numbers of LDs analysed in each lineage: 55±5 (E3.5 ICM); 54±4 (E3.5 Mural TE); 24±2 (E3.5 Polar TE); 66±31(E4.75 EPI); 14±3 (E4.75 PrE); 36±18 (E4.75 Polar TE); 305±28 (E4.75 Mural TE); 297±85 (E5.5 EPI); 548±110 (E5.5 EPI-VE); 59±18 (E5.5 ExE); 143±19 (E5.5 ExE-VE). Two-sided Mann-Whitney U test, ***; $p<0.001$. Exact p-values: E3.5 ICM vs E4.75 EPI, $p=1x10^{-30}$; E3.5 ICM vs E5.5 EPI, $p=8.4x10^{-44}$. See also Fig. 2j showing the same data in the developing epiblast lineage only. Source data are provided as a Source Data file.

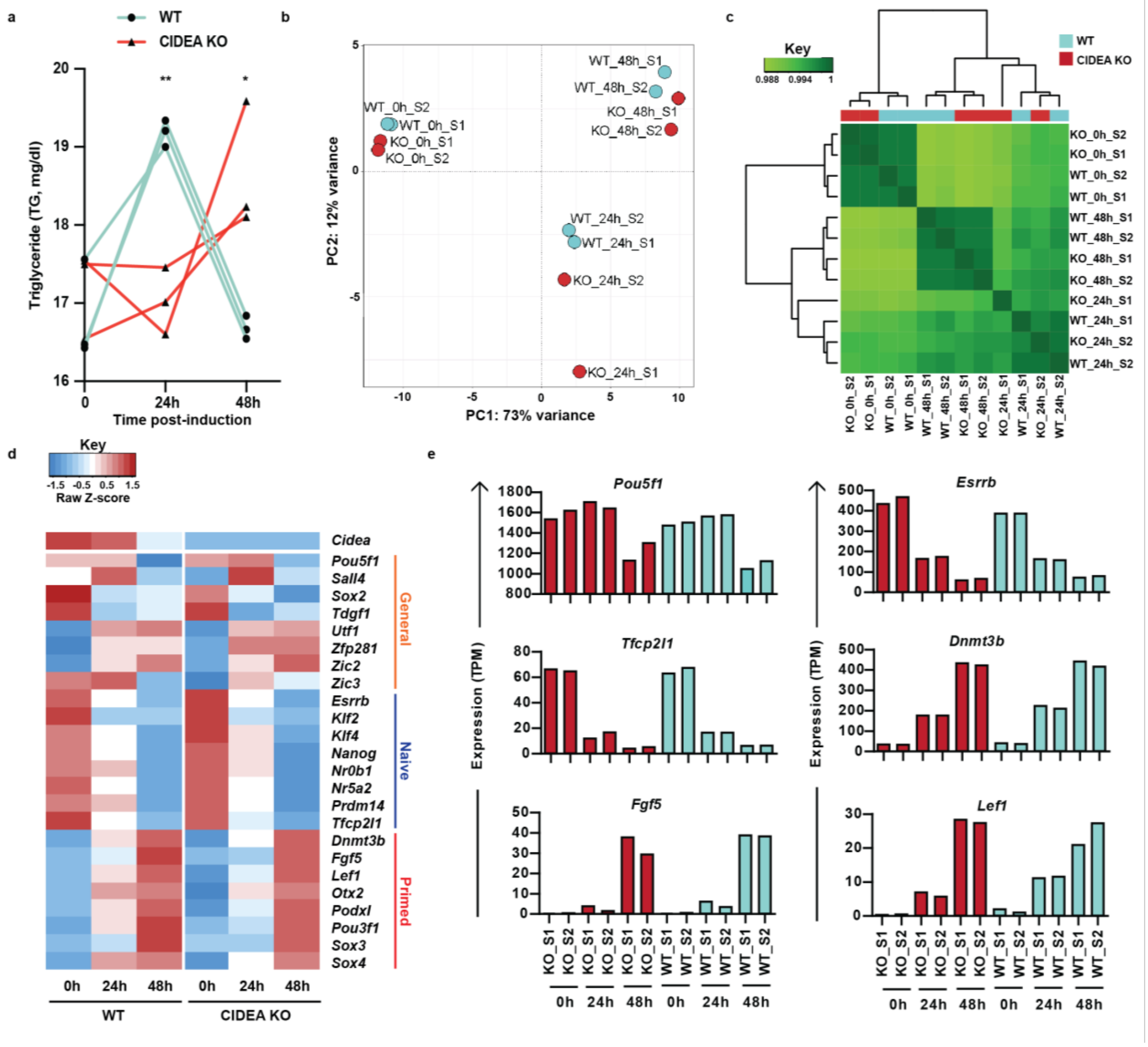

Supplementary Figure 5 | CIDEA knockout ESCs acquire normally a primed pluripotent state in 3D cultures. (a) Levels of cellular triglyceride (TG; mg/dl) in WT (blue) and CIDEA KO (red) ESCs prior to induction (0 hours) and post-induction (24, 48 hours) of spheroid differentiation. Measurements using a fluorometric-based assay were made on 1 x 10^6 cell equivalents as estimated by DNA content quantification (see Methods) (n=3). Two-sided unpaired Student's t-test, *, p<0.05; **, p<0.01. Exact p-values: 24h WT vs CIDEA KO, p=0.001243; 48h WT vs CIDEA KO, p=0.015271. **(b)** Principal Component Analysis of RNA-sequencing data collected from two independent samples (S1 and S2) of WT and CIDEA KO ESCs (clone 19.7) prior to induction (0 hours) and post-induction (24, 48 hours) of spheroid differentiation showing that the majority of variance is associated with the stages of differentiation along PC1 (n=2 biological replicates for each time point; each dot represents one sample). **(c)** Heatmap showing the correlation between expression profiles of RNA-sequencing samples across differentiation. Data are log-normalised, and correlation

was calculated using Pearson's Coefficient. **(d)** Average expression of selected general (orange), naive (blue) and primed (red) pluripotency genes in WT and CIDEA KO ESCs (0 hours) and spheroids (24, 48 hours) indicating that the transition from naive to primed pluripotency is not significantly affected upon loss of CIDEA. Scale bar represents log-normalised expression value. **(e)** Bar plots showing the expression of *Pou5f1, Esrrb, Tfcp2l1, Dnmt3b, Fgf5 and Lef1* as Transcript per Million (TPM) from RNA-sequencing datasets in (b-d). Source data are provided as a Source Data file.

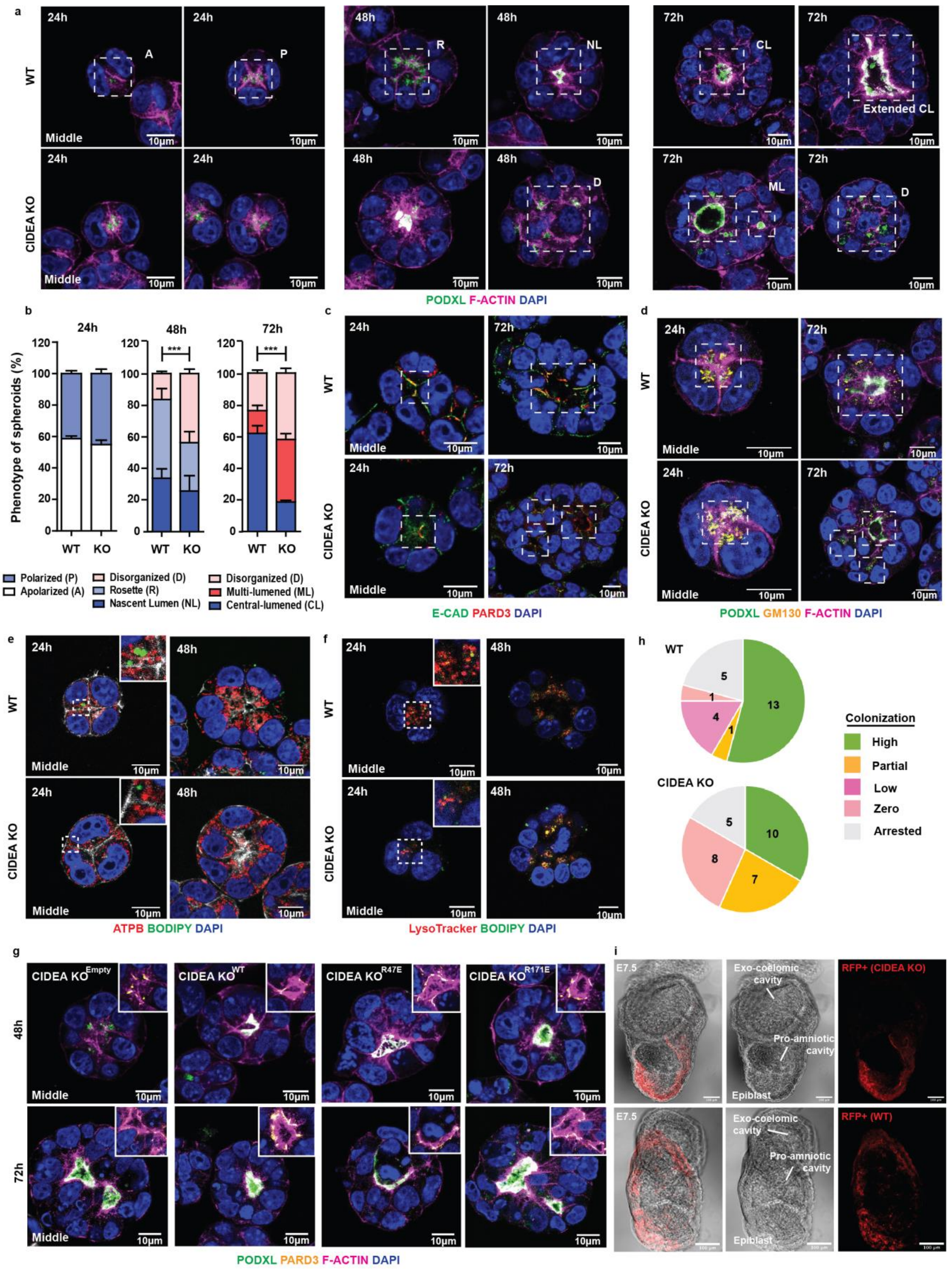

Supplementary Figure 6 | CIDEA knockout disrupts lumenogenesis in pluripotent cells *in vitro* and *in vivo*. **(a)** Formation of spheroids by WT and CIDEA KO ESCs over 72 hours of differentiation. Immunostaining against PODXL marks apical polarity (n=3). Phenotypes of spheroids are scored as follows: A, apolarized; P, polarized; R, rosette; NL, nascent lumen; CL, central-lumened; ML, multi-lumened; D, disorganized. **(b)** Quantification of the

phenotype of spheroids from (a) as defined (see also Fig. 3e showing the same data at 72 hours only). n ≥ 122 spheroids; error bars, means ± s.e.m. (n=3); two-sided Chi-square (Fisher exact) test; ***, p<0.001. Exact p-values: 48h WT vs KO, $p=3.5x10^{-6}$; 72h WT vs KO, $p=8.6x10^{-13}$. **(c,d)** Distribution of known polarity markers confirming the formation of multiple lumen foci in CIDEA KO spheroids 72 hours post-induction of differentiation. Apical membranes are marked by immunostaining against atypical PKC (aPKC) complex member PARD3 and adherens junction protein E-CADHERIN (E-CAD) (c). Golgi apparatus marker GM130 localized in sub-apical domains of polarized epithelium, along with cytoplasmic PODXL (d). (n=3) (**e,f**) Prevalent apical repositioning of mitochondria (marked by ATPB)(e) and lysosomes (labelled by LysoTracker Red)(f) is disrupted in CIDEA KO spheroids as imaged against BODIPY 493/503-stained LDs. Squares indicate magnified regions (n=3). **(g)** Representative images of spheroids formed by CIDEA KO ESCs stably transfected with empty (CIDEA KO^{Empty}), wild-type (CIDEA KO^{WT}) and point-mutated (CIDEA KO^{R47E} and CIDEA KO^{R171E}) V5-tagged *Cidea* constructs (n=3) (see also Supplementary Fig. 3). **(h)** Numbers of E5.5 embryo chimaeras showing high, partial, low or no epiblast colonization by injected LifeAct-RFP-expressing WT and CIDEA KO ESCs. 5/30 WT and 5/24 CIDEA KO embryo chimaeras were arrested in development. **(i)** Bright-field images of E7.5 embryo chimaeras highly colonized by LifeAct-RFP expressing WT and CIDEA KO ESCs, similarly showing a single, fully developed pro-amniotic cavity in agreement with the non-embryonic lethality of CIDEA KO mice[3] (n=2-3). Source data are provided as a Source Data file.

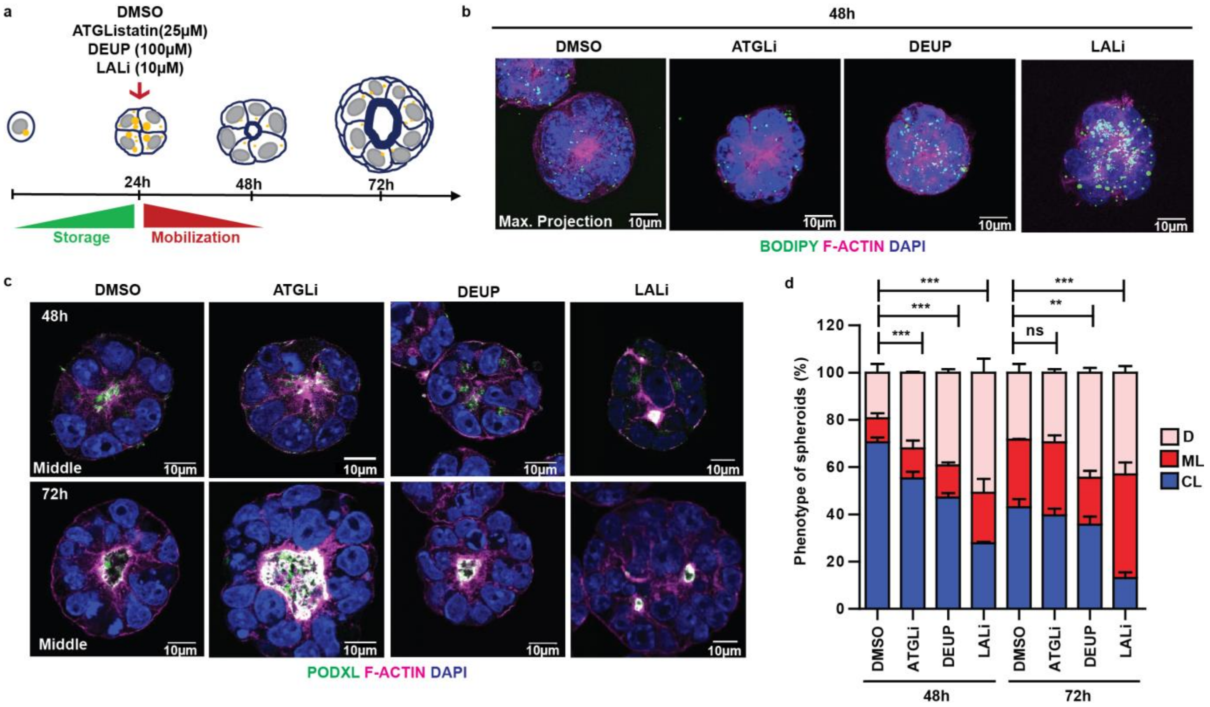

Supplementary Figure 7 | Lysosomal degradation of LDs is specifically required for spheroid morphogenesis. (a) Experimental set-up. Inhibitors selectively targeting the activity of the LD-associated lipase ATGL (ATGListatin), all cytosolic lipases (DEUP), lysosomal acid lipase (LAListatII) or vehicle control (DMSO) were added into spheroid cultures 24 hours post-induction of differentiation, and their effects analysed at indicated time-points. **(b)** Representative images of BODIPY 493/503-stained LDs in WT spheroids treated with DMSO, ATGListatin (ATGLi), DEUP or LAListatII (LALi) as outlined in (a) (n=3). **(c)** Representative images of spheroids formed under DMSO, ATGLi, DEUP or LALi treatment. Apical polarity is labelled by immunostaining against PODXL (n=3). **(d)** Quantification of the phenotype of spheroids from (c) (see also Fig. 5b showing the same data for DMSO or LALi treatment at 72 hours only). $n \geq 166$ spheroids; error bars, means ± s.e.m. (n=3); two-sided Chi-square (Fisher exact) test, ns, not significant; **, $p<0.01$; ***, $p<0.001$. Exact p-values: 48h DMSO vs 48h ATGLi, $p=0.0003$; 48h DMSO vs 48h DEUP, $p=9.5\times10^{-7}$; 48h DMSO vs 48h LALi, $p=2.7\times10^{-19}$; 72h DMSO vs 72h ATGLi $p=0.8106$; 72h DMSO vs 72h DEUP $p=0.0019$; 72h DMSO vs 72h LALi $p=5.6\times10^{-14}$. D, disorganised; ML, multi-lumened; CL, central-lumened. Source data are provided as a Source Data file.

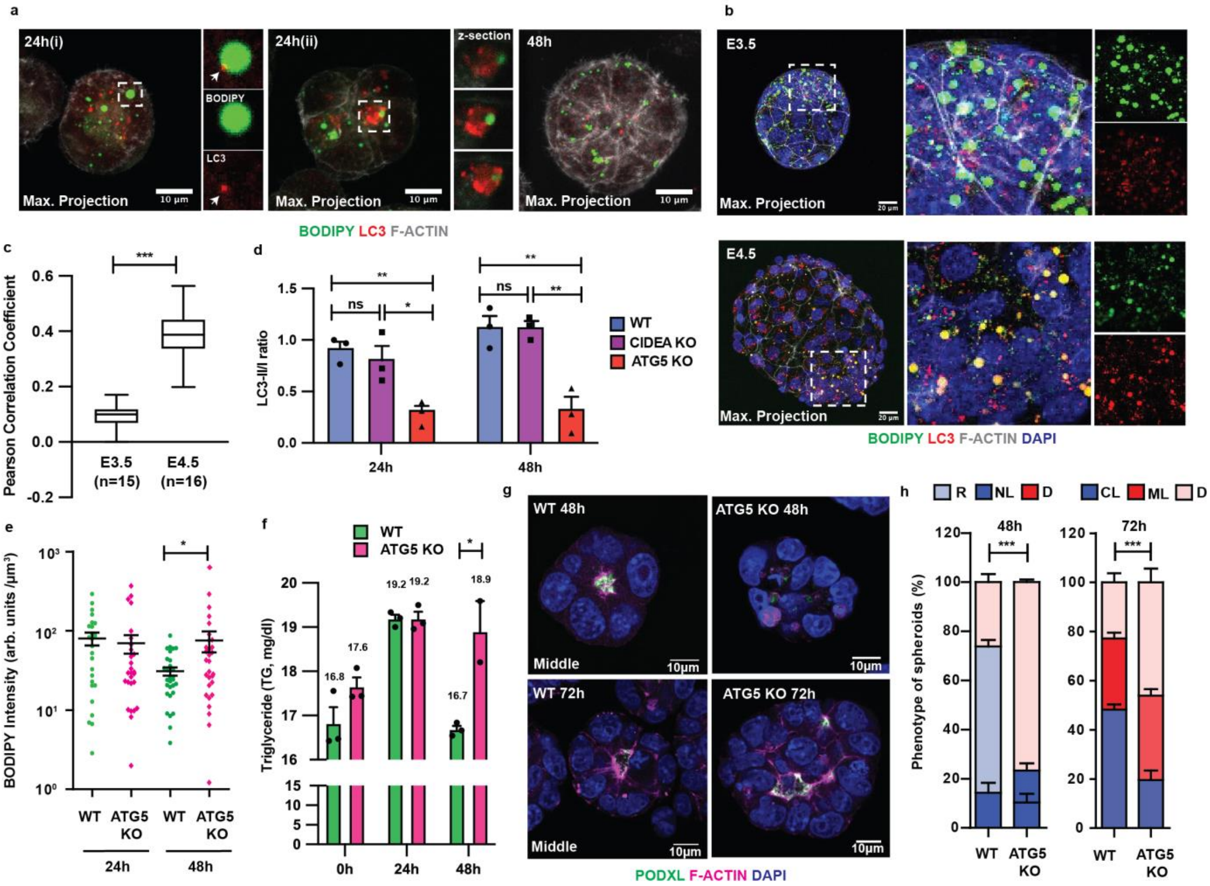

Supplementary Figure 8 | Lipophagy coordinates the timely mobilization of LDs and morphogenesis during peri-implantation development. (a) Representative images of BODIPY 493/503-stained LDs tagged (i) and engulfed (ii) into autophagosomes (marked by LC3) in WT ESC-induced spheroids at 24 hours post-induction of differentiation along with 48-hour WT spheroids shown for comparison (n=3). Squares indicate magnified regions. **(b)** Representative images of autophagosomes and LDs in E3.5 (~ 98 hours post-hCG injection) and E4.5 (~ 122 hours post-hCG injection) blastocysts stained as in (a) (n=15-16 blastocysts). **(c)** Co-localization analysis of LC3 and BODIPY signals in blastocysts from (b), showing autophagy degradation of LDs in late E4.5 blastocysts (n=15-16). Pearson Correlation Coefficient (+1, correlation to -1, anti-correlation); two-sided Mann-Whitney U test; ***, p< 0.001 (p=0.0005). Box limits = 25th-75th percentiles; centre lines = medians; whiskers end = min. and max. values. See also Fig. 5d,e showing comparable analysis upon staining for lysosomes (LysoTracker) and LDs. **(d)** Autophagy flux represented by LC3-II/I ratio as estimated by Western blotting (n=3) in spheroids formed by WT, CIDEA KO and ATG5 KO ESCs. Two-sided unpaired Student's t-test; ns, not significant; *, p<0.05; **, p<0.01. Exact p-values: 24h WT vs 24h CIDEA KO, p=0.53; 24h WT vs 24h ATG5 KO p=0.0036; 24h CIDEA KO vs 24h ATG5 KO, p=0.0312; 48h WT vs 48h CIDEA KO p=0.99; 48h WT vs

48h ATG5 KO p=0.0083; 48h CIDEA KO vs ATG5 KO, p=0.0044. **(e)** Quantification of neutral lipid content using BODIPY 493/503 staining (BODIPY fluorescence intensity normalised to spheroid volume) in spheroids formed by WT (green) and autophagy deficient ATG5 KO (magenta) ESCs[4] at indicated time-points. n=30 spheroids; error bars, means ± s.e.m. (n=3); two-sided Mann-Whitney U test, *, $p<0.05$ (p=0.0364). **(f)** Levels of cellular triglyceride (TG; mg/dl) in WT and ATG5 KO ESCs prior to induction (0 hours) and post-induction (24, 48 hours) of spheroid differentiation. Measurements using a fluorometric-based assay were made on 1×10^6 cell equivalents (n=3). Two-sided unpaired Student's t-test; *, $p<0.05$ (p=0.024). **(g)** Representative images of spheroids formed by WT and ATG5 KO ESCs at indicated time-points (n=3). **(h)** Quantification of the phenotype of spheroids from (g) (see also Fig. 5h showing the same data at 72 hours only). n ≥ 159 spheroids; error bars, means ± s.e.m. (n=3); two-sided Chi-square (Fisher exact) test; ***, $p<0.001$ (48h, $p=1.9\times10^{-20}$; 72h, $p=1\times10^{-8}$). R, rosette; NL, nascent lumen; D, disorganized; ML, multi-lumened; CL, central-lumened. Source data are provided as a Source Data file.

Supplementary References

1 Price PJ, G. M., Tilkins ML. Embryonic stem cell serum replacement. *International Patent Application WO 98/30679* (1998).

2 Barneda, D. *et al.* The brown adipocyte protein CIDEA promotes lipid droplet fusion via a phosphatidic acid-binding amphipathic helix. *Elife* **4**, e07485, doi:10.7554/eLife.07485 (2015).

3 Zhou, Z. *et al.* Cidea-deficient mice have lean phenotype and are resistant to obesity. *Nat Genet* **35**, 49-56, doi:10.1038/ng1225 (2003).

4 Mizushima, N. *et al.* Dissection of autophagosome formation using Atg5-deficient mouse embryonic stem cells. *J Cell Biol* **152**, 657-668, doi:10.1083/jcb.152.4.657 (2001).
