# Supplementary material for: Dynamic enlargement and mobilization of lipid droplets in pluripotent cells coordinate morphogenesis during mouse peri-implantation development
Source: Nat Commun. 2022 Jul 5;13:3861. doi: 10.1038/s41467-022-31323-2 (PMC9256688; doi:10.1038/s41467-022-31323-2)
Supplement: Supplementary file 3 — Description of Additional Supplementary Files [file 41467_2022_31323_MOESM3_ESM.pdf]

## Description of Additional Supplementary Files

File name: Supplementary Data 1

Description: Lists of oligonucleotides, antibodies and inhibitors used in this study.

File name: Supplementary Movie 1

Description: Timelapse movie of LDs in E14-ESCs. BODIPY-stained LDs underwent fusion in ESCs cultured in KSR-supplemented medium. Time-lapse Z-stacks were acquired every 2 minutes and represented as maximal projection (see Fig. 2b).

File name: Supplementary Movie 2

Description: Z-stack images of E5.5 WT embryo chimaera. Z-stack images of a representative embryo chimaera generated from injecting wild-type (WT) LifeACT-RFP expressing ESCs into E2.5 embryo (see Fig. 3h; upper panel).

File name: Supplementary Movie 3

Description: Z-stack images of E5.5 CIDEA KO embryo chimaera. Z-stack images of a representative embryo chimaera generated from injecting CIDEA knockout (KO) LifeACT-RFP expressing ESCs into E2.5 embryo (see Fig. 3h; lower panel).

File name: Supplementary Movies 4-5

Description: Time-lapse movies of LDs in two representative WT ESC-induced spheroids. BODIPY-stained LDs were maintained and gradually trafficked towards the apical domains of the spheroids. Timelapse Z-stacks were acquired every 10 minutes and represented as maximal projection (see Fig. 4a, left panel).

File name: Supplementary Movie 6

Description: Timelapse movie of LDs in a representative CIDEA KO ESCinduced spheroid. BODIPYstained LDs disappeared within ~2hours of imaging in the absence of CIDEA expression. Time-lapse Zstacks were acquired every 10 minutes and represented as maximal projection (see Fig.4a; right panel).
