# Supplementary material for: Dynamic enlargement and mobilization of lipid droplets in pluripotent cells coordinate morphogenesis during mouse peri-implantation development
Source: Nat Commun. 2022 Jul 5;13:3861. doi: 10.1038/s41467-022-31323-2 (PMC9256688; doi:10.1038/s41467-022-31323-2)
Supplement: Supplementary file 11 — Reporting Summary [file 41467_2022_31323_MOESM11_ESM.pdf]

nature research

Corresponding author(s): Veronique Azuara

Last updated by author(s): 16.05.2022

Reporting Summary

Nature Research wishes to improve the reproducibility of the work that we publish. This form provides structure for consistency and transparency in reporting. For further information on Nature Research policies, see our Editorial Policies and the Editorial Policy Checklist.

Statistics

For all statistical analyses, confirm that the following items are present in the figure legend, table legend, main text, or Methods section.

n/a	Confirmed	
☐	☒	The exact sample size (n) for each experimental group/condition, given as a discrete number and unit of measurement
☐	☒	A statement on whether measurements were taken from distinct samples or whether the same sample was measured repeatedly
☐	☒	The statistical test(s) used AND whether they are one- or two-sided<br>*Only common tests should be described solely by name; describe more complex techniques in the Methods section.*
☒	☐	A description of all covariates tested
☐	☒	A description of any assumptions or corrections, such as tests of normality and adjustment for multiple comparisons
☒	☐	A full description of the statistical parameters including central tendency (e.g. means) or other basic estimates (e.g. regression coefficient) AND variation (e.g. standard deviation) or associated estimates of uncertainty (e.g. confidence intervals)
☐	☒	For null hypothesis testing, the test statistic (e.g. F, t, r) with confidence intervals, effect sizes, degrees of freedom and P value noted<br>*Give P values as exact values whenever suitable.*
☒	☐	For Bayesian analysis, information on the choice of priors and Markov chain Monte Carlo settings
☒	☐	For hierarchical and complex designs, identification of the appropriate level for tests and full reporting of outcomes
☐	☒	Estimates of effect sizes (e.g. Cohen's d, Pearson's r), indicating how they were calculated

Our web collection on statistics for biologists contains articles on many of the points above.

Software and code

Policy information about availability of computer code

Data collection

Real-time qPCR: StepOne plus (Applied Biosystems)
Western Blot: GE ImageQuant LAS4000 imager (Cytiva) and Fiji ImageJ (v1.53i)
RNA sequencing: Illumina HiSeq (2 x 150bp configuration)
Imaging: Leica SP5 Confocal, Zeiss LSM710 Confocal, ZEISS AxioObserver Z1
Fluorescence plate reader: PHERAstar PLUS (BMG-Labtech)

Data analysis

RNA sequencing: Quality filtering of paired-end reads by FastQC; data trimming by Trimmomatic-0.33; quantification by Kallisto v0.43.1; subsequent analysis in R (version 4.0.2) using Bioconductor packages.

Imaging data: minor and full-image adjustment of brightness/contrast by ImageJ (v1.53i).
ImageJ package "3D fast filter" for minimizing background signal before quantification - see also Ollion, J. et al. TANGO: A Generic Tool for High-throughput 3D Image Analysis for Studying Nuclear Organization. Bioinformatics 29(14), 1840-1 (2013).
ImageJ package "EzColocalization" for measuring signal co-localization - see also Stauffer, W., Sheng, H. & Lim, H.N. EzColocalization: An ImageJ plugin for visualizing and measuring colocalization in cells and organisms. Sci Rep 8, 15764, doi.org/10.1038/s41598-018-33592-8 (2018).
ImageJ built-in "3D object counter" for quantifying signals.

Statistical analysis: GraphPad Prism version 5.01

For manuscripts utilizing custom algorithms or software that are central to the research but not yet described in published literature, software must be made available to editors and reviewers. We strongly encourage code deposition in a community repository (e.g. GitHub). See the Nature Research guidelines for submitting code & software for further information.

Data

Policy information about availability of data

All manuscripts must include a data availability statement. This statement should provide the following information, where applicable:

- Accession codes, unique identifiers, or web links for publicly available datasets
- A list of figures that have associated raw data
- A description of any restrictions on data availability

The RNA-sequencing data generated in this study have been deposited in the Gene Expression Omnibus database under accession code GSE165563. The RNA-sequencing data of mouse embryo development used in this study are available in the ArrayExpress database under accession code E-MTAB-2958.

Source data are provided with this paper, including RT-qPCR data (Fig. 2c; Fig. 3b; Supplementary Fig. 1c,d,f,g; Supplementary Fig. 2a,f,g,h), quantification of immunofluorescence data (Fig. 1b,c; Fig. 2e,f,j; Fig. 3c,d; Fig. 4c; Fig. 5a,e; Supplementary Fig. 4d; Supplementary Fig. 8c,e), records of spheroid phenotypes (Fig. 3e,f; Fig. 4e; Fig. 5b,h; Supplementary Fig. 6b; Supplementary Fig. 7d; Supplementary Fig. 8h), triglyceride assay (Supplementary Fig. 5a; Supplementary Fig. 7f), growth and self-renewal assays (Supplementary Fig. 2i,j,k,l) and uncropped western blots (Supplementary Fig. 2b; Supplementary Fig. 7d).

Time-lapse movies described in this study are available as Supplementary Movie 1 (Fig. 2b) and Supplementary Movies 4-6 (Fig. 4a). Z-stack images of representative highly colonized WT and CIDEA KO E5.5 embryo chimaeras are also provided as Supplementary Movies 2,3 (Fig. 3h).

Field-specific reporting

Please select the one below that is the best fit for your research. If you are not sure, read the appropriate sections before making your selection.

☒ Life sciences ☐ Behavioural & social sciences ☐ Ecological, evolutionary & environmental sciences

For a reference copy of the document with all sections, see nature.com/documents/nr-reporting-summary-flat.pdf

Life sciences study design

All studies must disclose on these points even when the disclosure is negative.

Sample size	Experimental design followed common standards in the field. No statistical methods were used to predetermine sample size. 24 and 30 mouse ESC-embryo chimaeras (E5.5) were generated for each cell type (WT and CIDEA KO, respectively) from which 13 (WT) and 10 (KO) were selected as highly colonized chimaeras for further analysis.
Data exclusions	No data were excluded from the analysis (unless clearly indicated in Source Data files).
Replication	All experiments including quantification experiments were performed in triplicate unless specified in Figure Legends. For example, experiments for embryo chimaeras were repeated three times and results were pooled together.
Randomization	Randomization is not a standard in the field.
Blinding	Blinding is not a standard in the field. On occasions, analyses were double-checked by different investigators.

Reporting for specific materials, systems and methods

We require information from authors about some types of materials, experimental systems and methods used in many studies. Here, indicate whether each material, system or method listed is relevant to your study. If you are not sure if a list item applies to your research, read the appropriate section before selecting a response.

Materials & experimental systems

n/a	Involved in the study	
☐	☒	Antibodies
☐	☒	Eukaryotic cell lines
☒	☐	Palaeontology and archaeology
☐	☒	Animals and other organisms
☒	☐	Human research participants
☒	☐	Clinical data
☒	☐	Dual use research of concern

Methods

n/a	Involved in the study	
☒	☐	ChIP-seq
☒	☐	Flow cytometry
☒	☐	MRI-based neuroimaging

Antibodies

Antibodies used	Primary antibodies:

ATBP (ab14730 Abcam; lot number: GR3236125-1)
CIDEA (sc-8730-R Santa Cruz; lot number: K1209)
CIDEA (13170-1-AP Proteintech; lot number: 00040898)
CDX2 (ab157524 Abcam; lot number: GR3181056-4)
E-CADHERIN (U3254 Sigma; lot number: 077M4800V)
ESRRB (H6705 R&D; clone number:H6705; lot number: A-2)
GM130 (CB1008 CalBiochem; lot number: 7069938)
OCT4 (ab27985 Abcam; lot number: GR3183882-3)
PARD3 (07-330 Millipore; lot number: 2987401)
RFP (ab62341 Abcam; lot number: GR3231879-2)
PODXL (MAB1556 R&D; clone number: 192703; lot number: IPF0320091)
V5 (46-0705 Invitrogen)
Vinculin (V9264 Sigma; lot number: 047M4795)
LC3 (ab192890 Abcam; clone number: EPR18709; lot number: GR3338049-2)
YAP (sc-101199 Santa Cruz; lot number: D0415)

Secondary antibodies:

Alexa633 Goat Anti-Mouse IgG (A21052 ThermoFisher)
Alexa633 Goat Anti-Rat IgG (A21094 ThermoFisher)
Alexa633 Donkey Anti-Goat IgG (A21082 ThermoFisher)
Alexa633 Goat Anti-Rabbit IgG (A21071 ThermoFisher)
Alexa555 Donkey anti-Mouse IgG (A31570 Invitrogen)
Alexa555 Donkey anti-Rabbit IgG (A32794 Invitrogen)
AlexaPlus647 Donkey anti-Rabbit IgG (A32795 Invitrogen)
Mouse anti-rabbit IgG-HRP (sc-2357 Santa Cruz)
Anti-Mouse IgG-HRP (sc-516102 Santa Cruz)

Validation

Antibodies were chosen based on previous literature. Description of the validation of primary antibodies listed above and any validation statements made by the manufacturers are accessible on the manufacturer's websites and relevant citations using the catalogue number of each antibody.

Eukaryotic cell lines

Policy information about cell lines

Cell line source(s)

Mouse wild-type R1 embryonic stem cells were gifted by Niall Dillon.
Mouse wild-type E14 embryonic stem cells were gifted by Austin Smith.
Mouse Atg5 KO and control embryonic stem cells were provided by Noboru Mizushima under MTA agreement - see reference Mizushima N et al., Dissection of autophagosome formation using Atg5-deficient mouse embryonic stem cells. J. Cell Bio. 152, 657-668 (2001).
CIDEA OE and control embryonic stem cells were generated from E14 wild-type cell line by stable transfection with empty (control) and V5-tagged Cidea (V5-CIDEA) constructs for this study.
CIDEA KO embryonic stem cells were generated from R1 wild-type cell line by CRISPR-Cas9 editing for this study.

Authentication

These cell lines were authenticated based on different culture condition requirements and phenotypic validations (e.g RT-qPCR, PCR, western blot and imaging).

Mycoplasma contamination

Testing for mycoplasma contamination was routinely performed using MycoZap assay (Lonza).

Commonly misidentified lines
(See ICLAC register)

No commonly misidentified cell lines were used in the study.

Animals and other organisms

Policy information about studies involving animals; ARRIVE guidelines recommended for reporting animal research

Laboratory animals

Laboratory animals used in this study are of the species Mus musculus (CD1 and C57BL/6J strains). Mice were housed in a 12-hour dark and 12-hour light cycle. CD1 females were crossed with either C57BL/6J or CD1 males to obtain stage-specific embryos. For natural mating, sexually mature CD1 females were used (usually 6-week to 3-month-old). For superovulation, 5 international units (iu) Pregnant Mare Serum Gonadotrophin solution (Folligon, MSD Animal Health) was administered to 3–4-week-old females via intraperitoneal injection. After 2 days, 5 iu human Chorionic Gonadotrophin solution (hCG; Chorulon, MSD Animal Health) was injected, followed by crossing with males (usually 3-6-month up to 18-month-old).

Wild animals

No wild animals were used in the study.

Field-collected samples

No field collected samples were used in the study.

Ethics oversight

All experiments performed in the U.K. were approved by the Home Office (project licences to M.P. and S.S.) and have been regulated by the Animals (Scientific Procedures) Act 1986 Amendment Regulations 2012 following ethical reviews by the Animal Welfare and Ethical Review Body (AWERB) at Imperial London College and the Local Ethical Review Panel (LERP) at the Department of Physiology, Anatomy and Genetics of the University of Oxford. Animal procedures performed in France were carried out according to French

national rules on Ethics and Animal Welfare in the Animal Facility (IERP, INRAE, doi: 10.15454/1.5572427140471238E12, Jouy-en-Josas). This work was approved by the French Ministry of Higher Education, Research, and Innovation (n°15-78) and the local Ethical Committee (INRAE Jouy-en-Josas Centre).

Note that full information on the approval of the study protocol must also be provided in the manuscript.
