# Supplementary material for: Dynamic enlargement and mobilization of lipid droplets in pluripotent cells coordinate morphogenesis during mouse peri-implantation development
Source: Nat Commun. 2022 Jul 5;13:3861. doi: 10.1038/s41467-022-31323-2 (PMC9256688; doi:10.1038/s41467-022-31323-2)
Supplement: Supplementary file 12 — Source Data [file 41467_2022_31323_MOESM12_ESM.zip › Source Data File/Source Data_Western uncropped blots.pptx]

## Slide 1
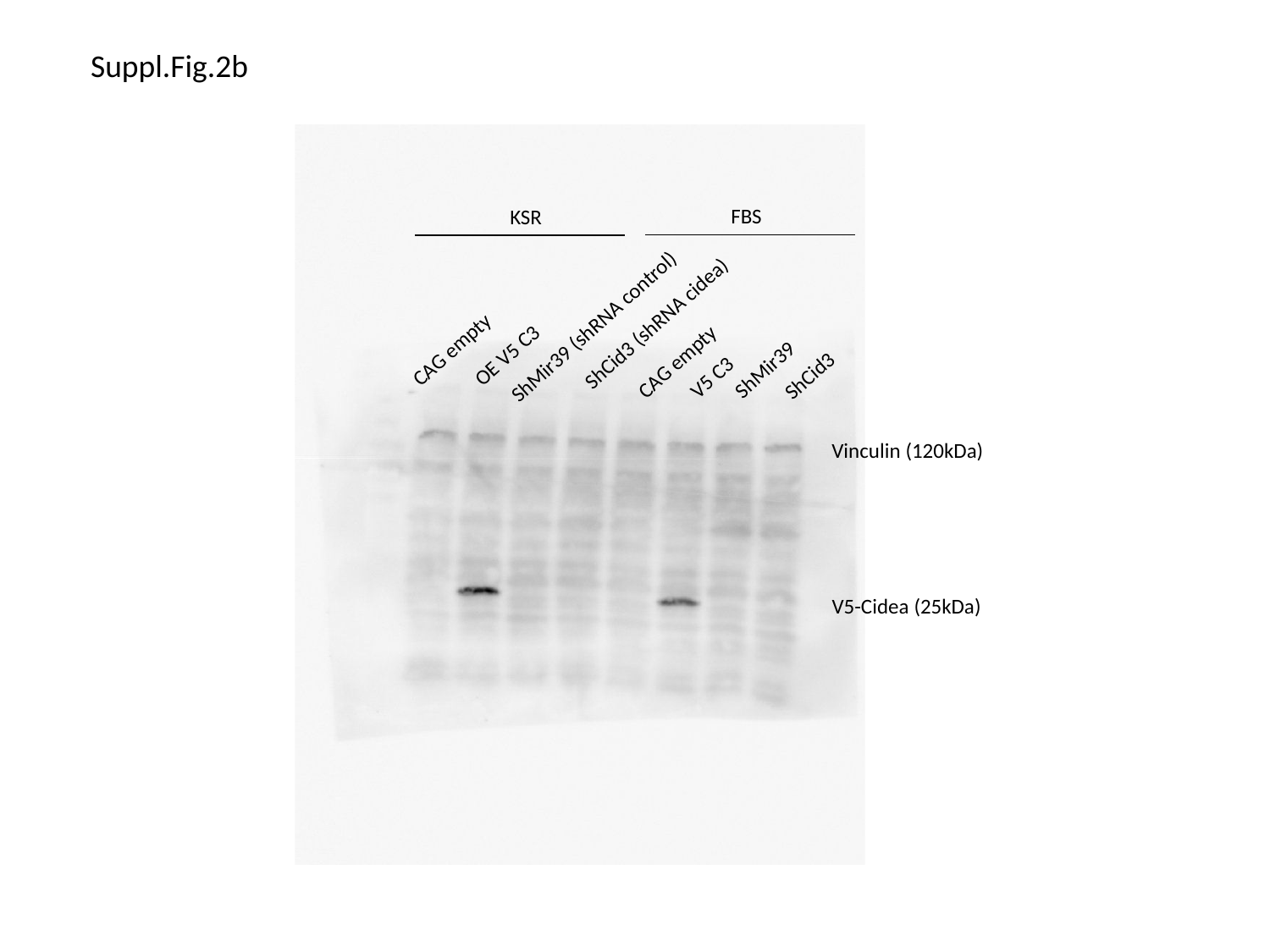

Suppl.Fig.2b
FBS
KSR
CAG empty
OE V5 C3
ShMir39 (shRNA control)
ShCid3 (shRNA cidea)
V5 C3
CAG empty
ShMir39
ShCid3
Vinculin (120kDa)
V5-Cidea (25kDa)

## Slide 2
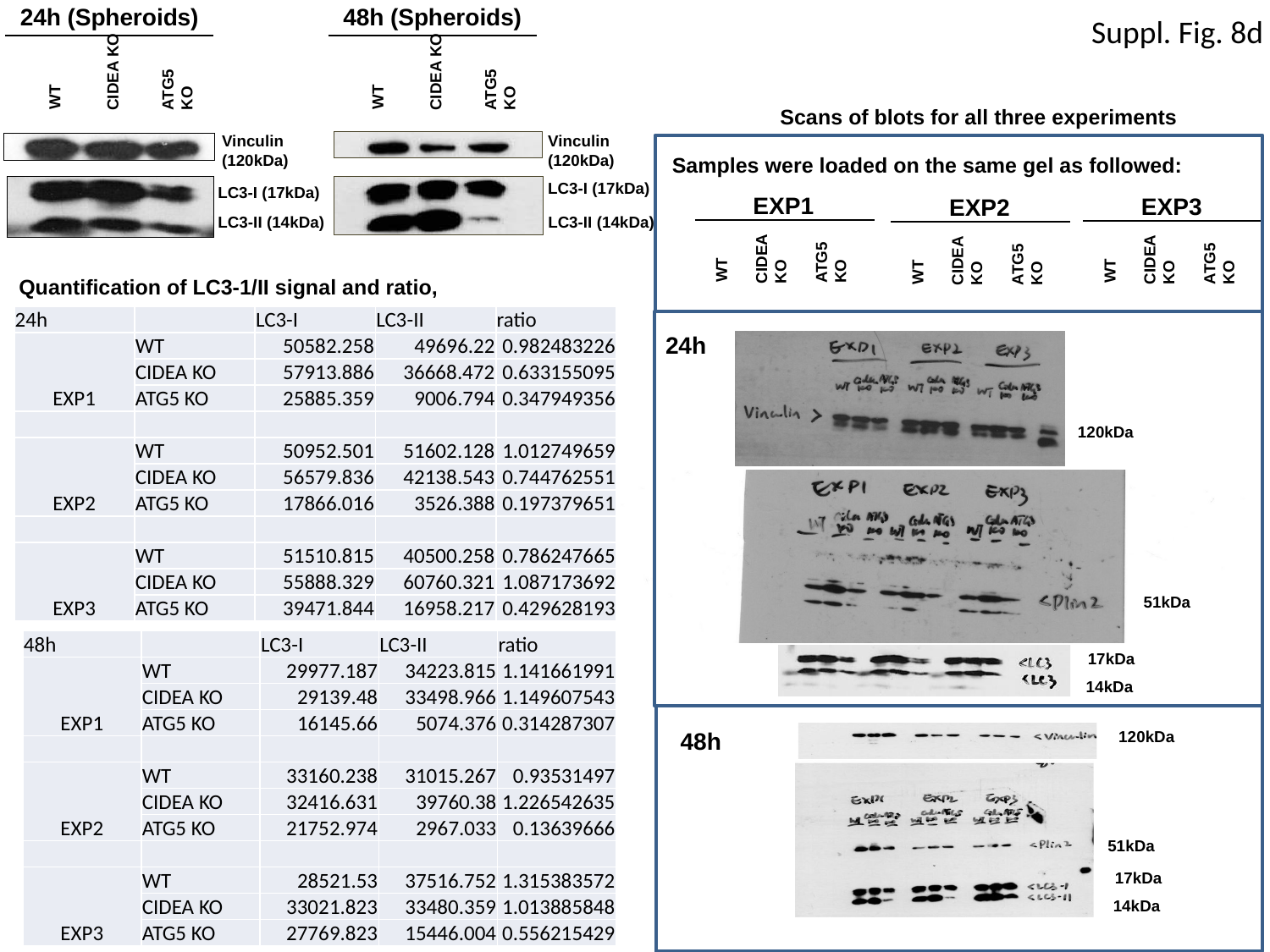

24h (Spheroids)
48h (Spheroids)
Suppl. Fig. 8d
CIDEA KO
CIDEA KO
ATG5 KO
ATG5 KO
WT
WT
Scans of blots for all three experiments
Vinculin
(120kDa)
Vinculin
(120kDa)
Samples were loaded on the same gel as followed:
LC3-I (17kDa)
LC3-I (17kDa)
EXP1
EXP3
EXP2
LC3-II (14kDa)
LC3-II (14kDa)
CIDEA KO
CIDEA KO
CIDEA KO
ATG5 KO
ATG5 KO
ATG5 KO
WT
WT
WT
Quantification of LC3-1/II signal and ratio,
| 24h | | LC3-I | LC3-II | ratio |
| --- | --- | --- | --- | --- |
| EXP1 | WT | 50582.258 | 49696.22 | 0.982483226 |
| | CIDEA KO | 57913.886 | 36668.472 | 0.633155095 |
| | ATG5 KO | 25885.359 | 9006.794 | 0.347949356 |
| | | | | |
| EXP2 | WT | 50952.501 | 51602.128 | 1.012749659 |
| | CIDEA KO | 56579.836 | 42138.543 | 0.744762551 |
| | ATG5 KO | 17866.016 | 3526.388 | 0.197379651 |
| | | | | |
| EXP3 | WT | 51510.815 | 40500.258 | 0.786247665 |
| | CIDEA KO | 55888.329 | 60760.321 | 1.087173692 |
| | ATG5 KO | 39471.844 | 16958.217 | 0.429628193 |
24h
120kDa
51kDa
| 48h | | LC3-I | LC3-II | ratio |
| --- | --- | --- | --- | --- |
| EXP1 | WT | 29977.187 | 34223.815 | 1.141661991 |
| | CIDEA KO | 29139.48 | 33498.966 | 1.149607543 |
| | ATG5 KO | 16145.66 | 5074.376 | 0.314287307 |
| | | | | |
| EXP2 | WT | 33160.238 | 31015.267 | 0.93531497 |
| | CIDEA KO | 32416.631 | 39760.38 | 1.226542635 |
| | ATG5 KO | 21752.974 | 2967.033 | 0.13639666 |
| | | | | |
| EXP3 | WT | 28521.53 | 37516.752 | 1.315383572 |
| | CIDEA KO | 33021.823 | 33480.359 | 1.013885848 |
| | ATG5 KO | 27769.823 | 15446.004 | 0.556215429 |
17kDa
14kDa
48h
120kDa
51kDa
17kDa
14kDa
